# Interpretable Adaptive Graph Fusion Network for Mortality and Complication Prediction in ICUs

**DOI:** 10.3390/diagnostics15222825

**Published:** 2025-11-07

**Authors:** Mehmet Akif Cifci, Batuhan Öney, Fazli Yildirim, Hülya Yilmaz Başer, Metin Zontul

**Affiliations:** 1 The Institute of Computer Technology, TU Wien University, 1040 Vienna, Austria; 2 Engineering and Informatics Department, Klaipėdos Valstybinė Kolegija/Higher Education Institution, 92294 Klaipeda, Lithuania; 3 Department of Computer Engineering, Bandırma Onyedi Eylül University, 10200 Balıkesir, Türkiye; 4 Department of Electronics and Automation, Vocational School, Istinye University, 34396 Istanbul, Türkiye; 5 Faculty of Economics, Administrative and Social Sciences, Department of Management Information Systems, Istanbul Topkapı University, 34087 Istanbul, Türkiye; 6 Department of Emergency Medicine, Faculty of Medicine, Bandirma Onyedi Eylul University, 10250 Balıkesir, Türkiye; 7 Department of Computer Engineering, Faculty of Engineering and Natural Sciences, Sivas University of Science and Technology, 58000 Sivas, Türkiye

**Keywords:** Adaptive Graph Fusion Network, electronic health records, intensive care, temporal modeling, interpretability, risk prediction

## Abstract

**Background:** This study introduces the Adaptive Graph Fusion Network, an interpretable graph-based learning framework developed for large-scale prediction of intensive care outcomes. The proposed model dynamically constructs patient similarity networks through a density-aware kernel that adjusts neighborhood size based on local data distribution, thereby representing both frequent and rare clinical patterns. **Methods:** To characterize physiological evolution over time, the framework integrates a short-horizon convolutional encoder that captures acute variations in vital signs and laboratory results with a long-horizon recurrent memory unit that models gradual temporal trends. The approach was trained and internally validated on the publicly available eICU Collaborative Research Database, which includes more than 200,000 admissions from 208 hospitals across the United States. **Results:** The model achieved a mean area under the receiver operating characteristic curve of 0.91 across six critical outcomes, with in-hospital mortality reaching 0.96, outperforming logistic regression, temporal long short-term memory networks, and calibrated Transformer-based architectures. Feature attribution analysis using SHAP and temporal contribution mapping identified lactate trajectories, creatinine fluctuations, and vasopressor administration as dominant determinants of risk, consistent with established clinical understanding while revealing additional temporal dependencies overlooked by existing scoring systems. **Conclusions:** These findings demonstrate that adaptive graph construction combined with multi-horizon temporal reasoning improves predictive reliability and interpretability in heterogeneous intensive care populations, offering a transparent and reproducible foundation for future research in clinical machine learning.

## 1. Introduction

Critical illnesses such as sepsis, respiratory failure, and acute kidney injury remain among the leading causes of death worldwide, accounting for nearly one-third of global mortality each year [1]. Patients admitted to intensive care units (ICUs) face particularly high risk, as complications like cardiac arrest and multi-organ failure often emerge suddenly and drive both early mortality and prolonged hospital stays [2,3]. Despite advances in patient monitoring, organ support, and clinical decision-support systems, accurately identifying those at imminent risk remains one of the central challenges in critical care [4].

The widespread adoption of electronic health records (EHRs) has created an opportunity to study disease progression at an unprecedented scale. EHRs combine demographic information, comorbidities, laboratory values, medication profiles, and continuous vital sign measurements, yielding a rich temporal map of each patient’s physiological state [5]. Numerous studies have shown that these data can support predictive models capable of anticipating deterioration hours before clinical recognition, potentially improving outcomes through earlier intervention [6]. However, real-world EHR data are far from clean. They are sparse, irregularly sampled, and composed of both static and time-varying variables. They also capture complex, nonlinear dependencies between patients and over-time patterns that conventional models struggle to represent reliably [7].

Traditional machine learning algorithms, such as gradient boosting and random forest ensembles, perform well on many structured data problems, including ICU mortality prediction [8,9]. They handle missing values and nonlinearity efficiently but rely heavily on feature engineering and cannot naturally model temporal evolution or patient–patient dependencies [10]. This limits their ability to adapt to rapidly changing clinical conditions.

Deep learning models alleviate some of these issues by learning representations directly from raw sequences. Transformer-based architectures have proven effective in modeling clinical event streams and capturing short-term dependencies within EHR data [11,12]. Antikainen et al. achieved mean AUCs above 75% for six-month mortality prediction using BERT and XLNet across more than 23,000 patients [13]. Yet, Transformers inherently assume a Euclidean structure and fixed sequence length, which fails to capture the non-Euclidean, relational organization of healthcare data [14]. Subsequent efforts—such as calibrated EHR Transformers [15] and long-horizon recurrent architectures with adaptive gating [16]—extend temporal depth but still treat each patient as an isolated sequence, ignoring inter-patient relationships that can inform prognosis.

Graph-based learning provides a natural way to model such relationships. By representing patients as nodes and their clinical similarities as edges, graph neural networks (GNNs) capture how information propagates within a population [17]. In healthcare, GNNs have been used to learn comorbidity networks, therapy–outcome associations, and patient similarity structures, often improving prediction accuracy and interpretability when compared with independent models. When combined with explainability tools such as SHapley Additive exPlanations (SHAP), these networks can highlight clinically meaningful predictors in ways that are understandable to physicians [18]. However, most existing GNN frameworks use *static* graphs if relationships between patients remain fixed throughout the hospital stay. This assumption is particularly problematic in ICUs, where patient states change minute by minute and the relevance of physiological variables evolves rapidly.

To address these challenges, we developed the Adaptive Graph Fusion Network (AGFN), an interpretable framework for ICU outcome prediction that integrates density-aware graph construction with multi-horizon temporal modeling and Shapley-based interpretability. Unlike static approaches, AGFN continuously updates the patient similarity graph as new measurements arrive, allowing relationships among patients to evolve with their physiological state. Short-horizon convolutional encoders capture acute fluctuations such as sudden drops in blood pressure or spikes in lactate, while long-horizon recurrent modules track slower trends such as renal decline or recovery from respiratory failure. These representations are fused through an attention-based graph operator that links temporal and relational information within a unified optimization process. The interpretability component implemented using SHAP and temporal contribution maps grounds the model’s predictions in clinical evidence, enabling transparent validation of the learned relationships.

Formally, let $G_{t}=\left(V,E_{t}\right)$ denote the time-varying patient graph, where each node i∈V corresponds to a patient with temporal feature vector $x_{i}(t)$. Edge weights are computed using a Gaussian kernel

(1) $$A_{t}(i,j)=exp\left(-\frac{{\left\|x_{i}(t)-x_{j}(t)\right\|}^{2}}{2\sigma^{2}}\right)$$

and local density is estimated as

(2) $$\rho_{i}(t)=\frac{1}{k_{0}}\sum_{j\in N_{k_{0}}(i)}\;A_{t}(i,j)$$

Here, σ denotes the kernel bandwidth controlling the smoothness of the Gaussian similarity function, and $\rho_{i}(t)$ represents the local density around patient i. The scaling factor h used in kernel density estimation adjusts the sensitivity of $\rho_{i}(t)$ to sparse regions, and Z is a normalization constant ensuring that the sum of all edge weights $\sum_{j}A_{t}(i,j)$ equals 1. The adaptive neighborhood size $k_{i}(t)$ scales inversely with $\rho_{i}(t)$, ensuring that patients in low-density regions—representing rare phenotypes—receive broader relational support. Temporal features are propagated across these evolving neighborhoods through an attention-based message-passing operator that learns contextual embeddings $h_{i}(t)$ combining individual dynamics and population structure. The resulting embeddings are decoded through a multi-task output layer to predict six ICU outcomes, including in-hospital mortality, sepsis, acute kidney injury, and prolonged length of stay.

We evaluated AGFN on the elCU Collaborative Research Database, which includes more than 200,000 ICU admissions from 208 hospitals across the United States. The model achieved a mean ROC-AUC of 0.91 across six clinical outcomes and 0.96 for mortality prediction, outperforming established baselines such as logistic regression, LSTM networks, and calibrated Transformer models. SHAP analysis identified lactate trajectories, creatinine dynamics, and vasopressor administration as key determinants of risk findings consistent with clinical literature, while revealing temporal patterns that existing scoring systems fail to capture. These results demonstrate that dynamic graph construction combined with multi-horizon temporal reasoning can significantly improve both robustness and interpretability in large-scale critical care modeling.

**Figure 1:**
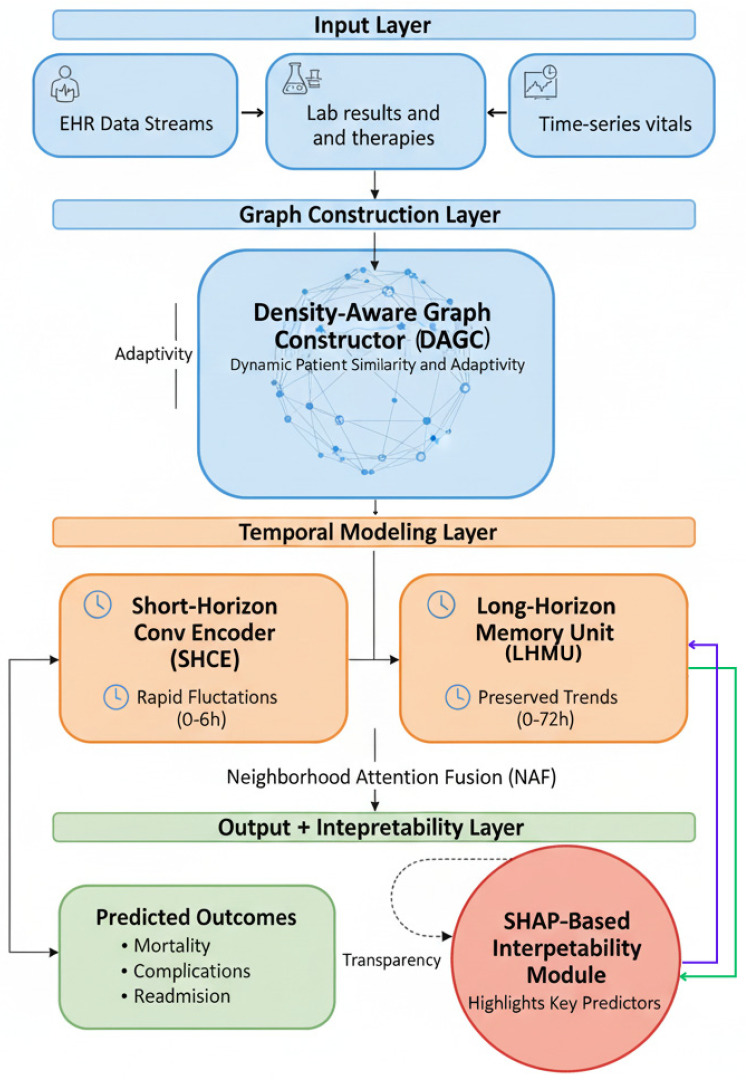
Overview of the proposed AGFN framework for interpretable ICU outcome prediction.

Figure 1 illustrates the core architecture of the proposed AGFN framework. It integrates data preprocessing, density-adaptive K-nearest-neighbor (DA-KNN) graph construction, short-term convolutional (ST-CNN) and long-term recurrent (LT-GRU) encoders for temporal modeling, and a SHAP-based interpretability module that links predictions to clinically relevant features.

## 2. Literature Work

The prediction of adverse ICU outcomes has long relied on risk scores such as TIMI and GRACE [19]. These LR-based indices use a limited set of predefined variables and remain interpretable but impose linearity assumptions that cannot capture the nonlinear dependencies typical of heterogeneous EHR data. With the growing availability of large-scale EHRs, research has shifted toward ML and DL frameworks capable of modeling high-dimensional, multimodal data without handcrafted feature engineering.

Classical ML algorithms, including SVM, RF, and NB, have been widely used for ICU prediction. These methods show strong discriminative power on structured EHR tables but depend on extensive preprocessing and feature selection. Tree-based ensembles, particularly XGB, have outperformed simpler ML models when paired with RFE or wrapper-based optimization. In [20], RFE-XGB achieved ACC = 91.5%, F1 = 95.1%, and AUC = 78.7%. In [21], class imbalance was mitigated through SMOTE and cost-sensitive learning, yielding AUC = 80.9% and MCC = 66.5% for minority outcomes. Variants such as LGBM and CB [22] have also been applied to ICU mortality and sepsis prediction, reaching high AUC values but relying on static admission features. For instance, ref. [23] reported AUC = 0.992 with CB, but the evaluation was restricted to static admission data and did not consider temporal dynamics. Although ensemble approaches highlight the predictive value of structured EHR data, they remain dependent on manual feature engineering and cannot model longitudinal trajectories, limiting generalizability in dynamic ICU cohorts [24].

DL frameworks overcome many of these limitations by learning hierarchical representations directly from raw EHR inputs. RNN-based models, especially LSTM and GRU, have been widely adopted for sequential prediction [25]. These models effectively capture temporal dependencies in irregularly sampled clinical data but suffer from vanishing gradients and limited scalability over long horizons. Hybrid CNN–RNN architectures improve local feature extraction yet remain unstable under sparse or irregular sampling. Transformer-based frameworks introduced SA mechanisms that model global temporal relations without recurrence [26]. For example, ref. [27] applied BERT and XLNet to more than 23,000 EHRs, achieving AUCs of 75.5% and 76.0% for six-month mortality prediction. However, standard transformers assume uniformly sampled Euclidean input spaces that poorly approximate non-Euclidean relational structures found in clinical data. Recent variants, including calibrated transformers, long-horizon recurrent hybrids [28], and Mamba-style gating models [16], extend temporal coverage and improve focus on salient clinical events but still lack mechanisms for inter-patient relational reasoning.

AGFNs and GNN-based frameworks represent a major step forward by explicitly modeling patient relationships as graph structures. In healthcare, AGFNs have been applied to patient-similarity graphs, comorbidity networks, and temporal co-occurrence structures [29]. By representing each patient as a node and defining edges through clinical similarity metrics, AGFNs capture both local neighborhood effects and higher-order dependencies. For example, ref. [30] combined an AGFN with LSTM to predict heart failure, achieving ACC = 98.9%. Although this study demonstrated the value of relational learning, its reliance on static graph construction limited adaptability when patient trajectories diverged after interventions. Such rigidity is particularly problematic in ICU populations, where physiological states such as sepsis, AKI, or respiratory failure evolve rapidly in response to treatment. In our eICU-CRD analysis, patients initially clustered by admission features often diverged within 72 h due to sodium fluctuations, vasopressor titration, or ventilatory deterioration. This dynamic heterogeneity highlights the need for graph construction strategies that adapt continuously to evolving data distributions.

Recent graph–temporal hybrids have emerged to address these limitations. Gformer [31], GraphEHR [32], Temporal Graph Neural Networks (TGNNs) [33], and GraphTimeNet [34] combine graph reasoning with temporal attention to capture both evolving topology and sequential context. These frameworks achieve state-of-the-art results in EHR-based forecasting but often retain fixed neighborhood definitions or uniform time windows, limiting adaptability in heterogeneous ICU cohorts. The proposed AGFN extends this paradigm by introducing density-adaptive graph construction (DA-KNN) and dual-scale temporal modeling. The short-term CNN (ST-CNN) captures acute physiological changes, while the long-term GRU (LT-GRU) with Mamba-inspired gating preserves chronic dependencies. This architecture allows stable information propagation across adaptive neighborhoods while maintaining temporal interpretability.

Interpretability remains a decisive factor for clinical deployment. Even highly accurate DL models face skepticism if their decision processes are opaque. SHAP provides model-agnostic interpretability through additive feature attributions derived from cooperative game theory [35]. In cardiovascular prediction, it was demonstrated that SHAP [36] accurately identified AGE and HTN as dominant predictors, validating clinical plausibility. Within ICU prognosis, SHAP analysis has revealed temporal patterns such as sodium slopes, lactate trajectories, and respiratory-rate variability that correspond closely to physician judgment yet are often neglected in static risk scores. Integrating SHAP with GNN–temporal architectures bridges algorithmic prediction and clinical reasoning, enhancing transparency and trust.

Despite advances in ML, DL, and AGFNs, several key challenges remain unresolved. First, static relational modeling underrepresents the evolving nature of ICU populations, where patient similarity graphs shift rapidly due to interventions and complications. Second, temporal scale limitations persist: CNNs capture local fluctuations, while RNNs and transformers capture longer horizons, but few frameworks unify both without sacrificing stability or scalability. Third, multimodal integration remains underdeveloped. EHRs contain demographics, diagnoses, labs, vitals, treatments, and narratives, each with distinct noise and missingness patterns. Finally, systematic interpretability is often an afterthought rather than a core design feature, creating a gap between predictive accuracy and clinical usability.

AGFN addresses these gaps by integrating adaptive graph construction, multi-scale temporal modeling, and built-in interpretability into a unified architecture. DA-KNN dynamically recalibrates neighborhood size using KDE, preserving patient similarity in heterogeneous cohorts while avoiding oversmoothing. ST-CNN modules capture acute fluctuations such as abrupt hemodynamic changes, while LT-GRU with Mamba-inspired gating models sustained dependencies like progressive renal decline. Multi-head attention propagates information across adaptive neighborhoods, and SHAP analysis provides transparency by attributing predictions to clinically meaningful features such as age, sodium dynamics, and vasopressor use. This design directly responds to the limitations of prior ML, DL, and AGFN approaches, enabling adaptive, interpretable, and deployment-ready outcome prediction in ICU populations.

## 3. Methods and Dataset

### 3.1. Overview

The proposed AGFN framework (Algorithm 1) predicts intensive care outcomes by combining adaptive graph construction, multi-scale temporal modeling, and interpretable learning. The process begins with structured preprocessing of EHRs where heterogeneous variables are normalized and missing values are addressed using a principled imputation strategy. These standardized features form the foundation for a DA-KNN module that constructs evolving patient–patient graphs. By adjusting neighborhood size according to local data density, the module captures clinically meaningful relationships that static graphs often overlook, such as similarities between rare but high-risk patient subgroups.

Temporal evolution is captured through two coordinated encoding branches. The ST-CNN focuses on rapid physiological fluctuations, including abrupt changes in heart rate, mean arterial pressure, oxygen saturation, and key laboratory indicators. In contrast, the LT-GRU, enhanced with a Mamba-inspired gating mechanism, models sustained temporal dependencies that span the full duration of an ICU stay. Combining these encoders yields feature representations sensitive to both acute events and gradual disease trajectories. The resulting embeddings are processed by GraphTransformer layers, which integrate temporal and relational information using attention mechanisms that highlight clinically relevant interactions rather than uniformly weighting all connections.

To ensure clinical transparency, the framework employs SHAP to decompose each model prediction into individual feature contributions. This analysis consistently identifies factors such as age, serum sodium trends, vasopressor adjustments, and respiratory variability as dominant predictors. These findings align with established clinical reasoning, demonstrating that AGFN not only achieves high predictive accuracy but also provides interpretable insights that can support real-time decision-making in critical care.
**Algorithm 1. Adaptive Graph Fusion Network Training and Evaluation**→Input: Preprocessed EHR sequences $X={{\left\{x_{1}(t)\right\}}_{i=1}\;}^{N}$, outcome labels $Y={{\left\{y_{1}\right\}}_{i_{=1}}\;}^{N}$, number of folds K→Output: Trained AGFN model $\Theta^{*}$ with calibrated predictions and interpretability maps→*Initialization:*→Set hyperparameters $\left\{k\_\right.$base, $\left.r,h,a\right\}$; fix random seed for reproducibilityFor f=1 to K do*Patient-Level Stratified Partitioning* →Split patients into ${D\_\mathrm{train}\;}^{\left(f\right)},\;{D\_\mathrm{val}\;}^{\left(f\right)},\;{D\_\mathrm{test}\;}^{\left(f\right)}$ with stratified sampling2.*Preprocessing (fitted on training data only)* →Fit robust scaler δ and exponential-smoothing imputation J on $D\_{\mathrm{train}\;}^{\left(f\right)}$ →Apply s and J to transform $D\_{\mathrm{val}\;}^{\left(f\right)}$ and $D\_{\mathrm{test}\;}^{\left(f\right)}$3.*Supervised Contrastive Embedding* →Train embedding network $\varphi_{\_}$emb on ${D\_\mathrm{train}\;}^{\left(f\right)}$ using →Obtain embeddings: $h_{i}=\varphi$_emb $\left(x_{i}\right)$4.*Density-Adaptive Graph Construction* →Compute local densities ρ via KDE →Calculate adaptive neighborhood sizes: $k_{i}=min$ →Construct DA-KNN graph $G_{t}=\left(V,E_{q}\right)$ with Gaussian edge weights →Freeze graph topology for validation/test5.*Dual-Scale Temporal Encoding* →*For each patient* ${i\in D\_train\;}^{f}$ *:* →ST-CNN: $y_{t}^{sT}=\sigma \left(W_{-}g*X_{tt_{t}v}\right)⊙\varphi \left(W_{-}c*X_{tt_{t}v}\right)$ →LT-GRU: ${h_{t}\;}^{LT}$ via Mamba gating →Concatenate: $H_{i}=\left[{h_{i}\;}^{ST};{h_{i}\;}^{LT}\right]$6.*GraphTransformer Integration* →Apply attention: $Attn(Q,K,V)=softmax\left(QK^{⊤}/\sqrt{d}\_k\right)V$ →Compute predictions: $\hat{y}=\sigma \left(W_{-}oH\_\right.$final $\left.+\;b_{-}o\right)$7.*Model Optimization* →Train Θ using AdamW with cosine annealing and gradient clipping →Early stopping based on validation AUC on ${D\_\mathrm{val}\;}^{\left(f\right)}$8.*Test Evaluation* →Evaluate on $D\_{\mathrm{test}\;}^{\left(f\right)}$ using frozen preprocessing and graph parameters →Record: ROC-AUC, AUPRC, precision, recall, F1-score, Brier score →End For9.*Cross-Validation Aggregation* →Compute mean metrics and 95% confidence intervals via bootstrap (1000 iterations)10.*Interpretability Analysis* →Apply SHAP for feature attributions: →Global feature importance rankings →Patient-level temporal contribution maps →Return: Model $\Theta^{*}$, performance metrics, SHAP interpretability maps

**Figure 2:**
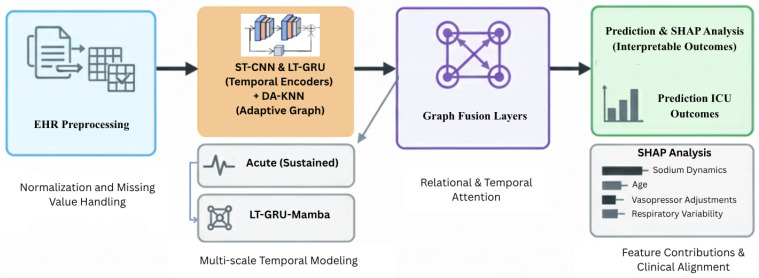
AGFN pipeline. Overview of the GraphTransformerNet workflow from EHR preprocessing through adaptive graph construction and dual-scale temporal encoding to SHAP-based interpretability.

Figure 2 illustrates the complete AGFN pipeline, beginning with EHR preprocessing for normalization and imputation. The processed features are used to construct a DA-KNN graph that captures dynamic patient relationships. Short-term CNN and long-term GRU encoders extract temporal patterns across multiple time scales, and their fused representations are forwarded to the GraphTransformer layers for integrated relational and temporal prediction.

### 3.2. Dataset and Feature Taxonomy

We conducted experiments on the eICU Collaborative Research Database v2.0 [34], which contains de-identified data from 200,859 ICU admissions across 208 hospitals in the United States. The eICU database, one of the largest public critical care datasets, captures diverse patient demographics, comorbidities, treatments, and outcomes across 208 U.S. hospitals. Such scale and heterogeneity make it particularly well suited for evaluating the robustness and generalizability of graph-based predictive models.

The feature space integrates both static descriptors and temporal sequences. Admission-level features capture demographic and baseline clinical information, including age, sex, body mass index, admission type, and comorbidity profiles such as diabetes mellitus, chronic kidney disease, and chronic obstructive pulmonary disease. The cohort has a mean age of 63.4 years with a standard deviation of 14.9, and 54.7 percent of patients are male.

Dynamic variables encode continuous monitoring throughout the ICU stay. Physiological measurements such as heart rate, mean arterial pressure, oxygen saturation, and respiratory rate are recorded at sub-hourly intervals, while laboratory values including lactate, creatinine, bilirubin, and arterial pH are measured less frequently, with a median sampling interval of approximately eight hours. Therapeutic interventions are also documented, capturing events such as mechanical ventilation, vasopressor use, and renal replacement therapy.

For outcome modeling, six binary endpoints were defined: in-hospital mortality, sepsis onset, acute kidney injury, respiratory failure requiring invasive mechanical ventilation, cardiac arrest, and prolonged ICU stay exceeding seven days. These endpoints encompass both acute catastrophic events and longer-term deterioration trajectories, directly linking model predictions to clinically meaningful risks and resource utilization.

The dataset exhibits heterogeneous missingness, reflecting real-world collection practices. Vital signs are nearly complete due to automated monitoring, while laboratory values such as lactate and bilirubin show sparsity exceeding 40 percent, as they are typically ordered only for patients suspected of organ dysfunction. Handling these irregularities requires imputation strategies that preserve both acute reactivity and long-term stability in temporal signals.

This feature taxonomy enables density-adaptive graph construction, where patient similarity relationships are informed jointly by baseline descriptors, evolving physiological signals, and intervention histories. Such representation captures the dual influence of chronic predisposition and acute temporal dynamics, allowing the model to identify clinically coherent subgroups, such as patients progressing toward septic shock or multi-organ failure.

Table 1 summarizes baseline cohort characteristics, including demographics, comorbidities, and intervention frequencies. These variables form the structured input space used for DA-KNN graph construction.

### 3.3. Preprocessing and Leakage Prevention

To mitigate temporal information leakage, we enforced strict censoring of features relative to outcome onset. For each complication, the exact timestamp of first occurrence was identified, and all features recorded after that time were excluded from training and inference for that patient–outcome pair. For example, if a patient experienced cardiogenic shock on day two, only admission features and day one laboratory trajectories were available to the model; all later measurements were masked. Sequence truncation ensured that imputation and derived features (slopes, deltas) were computed only from pre-outcome values. Intervention-related variables, such as fibrinolysis or drug administration, were similarly censored when recorded after an outcome event.

Leakage controls were applied within each cross-validation fold independently. Graph construction, kernel density estimation, and supervised contrastive embedding training were fit exclusively on the training patients in that fold, preventing any information from validation or test patients leaking through neighborhood definitions. Patient-level partitioning further guaranteed that no temporal records, derived embeddings, or graph edges crossed fold boundaries.

To verify these safeguards, we ran a control experiment in which post-outcome features were deliberately retained. This “leakage-allowed” model produced unrealistically high AUC values (>0.98), confirming that outcome-timestamp censoring was necessary. We also audited the feature-masking logs for random samples of patients, confirming that all records beyond outcome onset were correctly excluded.

#### 3.3.1. Outlier Mitigation

Continuous clinical variables exhibited occasional extreme values arising from measurement artifacts or rare pathological states. To prevent distortion of distributional properties while retaining statistical fidelity, we applied percentile-based winsorization at the 0.5th and 99.5th thresholds. The transformation is defined as:

(3) $${\hat{x}}_{i}=\left\{\begin{array}{ll} q_{0.005}, & x_{i}<q_{0.005}\\ q_{0.995}, & x_{i}>q_{0.995}\\ x_{i}, & otherwise\; \end{array}\right.$$

where ${\hat{x}}_{i}$ denotes the transformed value of the *i*-th observation, and q_p_ represents the *p*-th quantile of the empirical distribution.

This procedure reduced the influence of spurious laboratory results (for example, sodium levels beyond physiologically plausible ranges) while preserving clinically relevant variance. The resulting variables exhibited stabilized distributions, which enhanced the robustness of both graph construction and temporal modeling in subsequent pipeline stages.

#### 3.3.2. Missing Value Modeling

To address incomplete records, we explicitly modeled missingness using a binary indicator mask and applied a decay-aware exponential smoothing strategy for imputation. Clinical time-series are typically irregular and sparse, since laboratory values, for instance, are not collected at every time step. Naïve approaches such as mean imputation disregard temporal dynamics and may create unrealistic stability in patient trajectories. In contrast, exponential smoothing emphasizes recent measurements, which are generally more clinically informative, while reverting to the patient-specific baseline during extended gaps. This dual mechanism balances sensitivity to acute changes with robustness to chronic conditions, thereby generating imputations that more accurately reflect physiological evolution. The imputation rule is defined as

(4) $${\tilde{x}}_{t}=m_{t}x_{t}+\left(1-m_{t}\right)\left[\alpha {\tilde{x}}_{t-1}+(1-\alpha )\bar{x}\right],$$

where $m_{t}\in \{0,1\}$ denotes the missingness mask (1 if observed, 0 if missing), $x_{t}$ is the observed value when available, ${\tilde{x}}_{t-1}$ is the previously imputed value, $\bar{x}$ is the patient-specific mean, and α∈[0,1] is the decay coefficient controlling the trade-off between short-term recency and long-term baseline. This formulation ensures that imputations for consecutive missing values progressively decay toward stable patient-level statistics, mitigating bias from either over-reliance on recent noisy fluctuations or excessive smoothing toward global averages.

We selected exponential smoothing rather than multivariate imputation techniques such as MICE because ICU time series exhibit irregular and often informative missingness patterns. Exponential decay prioritizes recent observations, preserving temporal dynamics crucial for acute physiological changes, while avoiding the iterative regression overhead and distributional assumptions of MICE. This method supports real-time inference without model retraining and aligns with prior findings showing that time-aware decay mechanisms maintain higher fidelity in irregular clinical streams.

#### 3.3.3. Derived Temporal Features

To enrich the temporal representation of dynamic variables, we derived two complementary features: slope and stepwise difference. Clinical relevance lies not only in static values but in how they evolve. For example, a rising creatinine slope can indicate early kidney injury well before a single elevated measurement becomes critical, while short-term deltas capture acute events such as sudden drops in serum sodium. These derived features expose rate-of-change information that is not present in raw measurements. The slope quantifies long-range progression across an observation window:

(5) $$s=\frac{x_{t_{n}}-x_{t_{1}}}{t_{n}-t_{1}}$$

where $x_{t_{n}}$ and $x_{t_{1}}$ denote the values of the variable at the last and first timestamps, respectively.

The stepwise difference captures short-term variability between consecutive time points:

Δ = x_t_ − x_t−1_ (6)

Together, *s* highlights overall trends while Δ reflects abrupt fluctuations, enabling the model to capture both gradual shifts and acute changes in patient state.

#### 3.3.4. Scaling and Encoding

Medical data often exhibit heavy-tailed distributions, particularly lab measurements that can be skewed by rare but extreme values such as those observed in shock states. Standard z-scoring relies on mean and standard deviation, which are highly sensitive to such extremes. To mitigate this, we used a robust z-score transformation based on the median and interquartile range (IQR). This improves robustness by reducing the undue influence of outliers, ensuring that scaling reflects the central tendency of the population rather than rare pathological extremes. Continuous variables were thus standardized as:

(7) $$z_{i}\frac{x_{i}-median(x)}{IQR(x)}$$

where $x_{i}$ is observed value, median(x) centers the data around the median rather than the mean, making it less sensitive to outliers and IQR(x) scales the data using the middle 50% of the distribution, which also improves robustness. Categorical variables were transformed into binary vectors via one-hot encoding to ensure compatibility with downstream machine learning models.

### 3.4. Density-Adaptive Graph Construction

In our framework, each patient record is represented as a node in a dynamically evolving graph. The edges are determined through supervised contrastive embeddings, which ensures that clinically similar patients (with respect to outcomes and temporal trajectories) are positioned closer in the latent space. Clinical similarity in medicine is rarely about raw distance alone; two patients may appear close in feature space, but only outcome-aligned embeddings capture what is clinically meaningful. This design allows the graph to reflect medical proximities that matter for prediction, instead of relying on static distance metrics that can ignore label-dependent structure.

To avoid degenerated neighborhoods early in training, we anneal k_base_ from a small value to its target over the first 20% of epochs. We also cap per-node degree changes to Δk_i_ ≤ 2 between graph refreshes to prevent abrupt topology shifts.

#### 3.4.1. Embedding Generation

We first derived patient-level embeddings h_i_ by optimizing a supervised contrastive learning objective. Clinical similarity in medicine is often label-dependent. For example, two patients with the same ICU outcome (e.g., mortality or sepsis) should be embedded closer together than random neighbors. Simply using raw distance in feature space ignores outcome relevance. Supervised contrastive loss addresses this by enforcing task-driven similarity: embeddings of patients who share the same outcome cluster tightly, while embeddings of patients with different outcomes are explicitly separated. This ensures the learned representation reflects clinically meaningful relationships rather than just superficial feature overlap. Specifically, embeddings were trained to minimize:

(8) $$L_{\sup\text{-}\mathrm{contr}\;}=-\frac{1}{N}\sum_{i=1}^{N}\;\frac{1}{\left|P(i)\right|}\sum_{p\in P(i)}\;log\frac{exp\left(\frac{sim\left(h_{i},h_{p}\right)}{\tau }\right)}{\sum_{a=1}^{N}\;\;1_{\left[a\neq i\right]}exp\left(\frac{sim\left(h_{i},h_{a}\right)}{\tau }\right)}$$

The definition of the positive set P(i) is critical for clinical validity. For each prediction task, we define positives as all patients who share the same outcome label with patient *i*. In the multi-outcome setting, we adopt a one-vs-rest formulation: for a given outcome, patients experiencing that complication are treated as positives, while all others are negatives. This ensures that the embedding space captures outcome-specific similarity rather than collapsing across unrelated events. To counter class imbalance, we reweight positive pairs by the inverse prevalence of their associated outcome, preventing common complications (e.g., hypertension) from dominating the representation. This design allows embeddings to emphasize clinically rare but high-risk trajectories while maintaining balanced training dynamics.

To maintain full partition independence, the supervised contrastive encoder is retrained within each cross-validation fold using only training patients. Its weights are then frozen before embedding validation or test data, ensuring that no outcome labels, gradients, or latent representations from held-out partitions influence the embedding space. This fold-specific recomputation guarantees complete information isolation and prevents any leakage during graph construction or evaluation.

#### 3.4.2. Adaptive Neighborhood Size

A fixed neighborhood size kkk is insufficient to capture the heterogeneity of patient distributions. Using a constant kkk across the entire cohort leads to under-connection in sparse regions, where rare phenotypes may otherwise become isolated, and over-connection in dense regions, where redundant edges can dilute meaningful similarity signals. To overcome this limitation, we introduce an adaptive neighborhood size strategy that dynamically adjusts connectivity based on local embedding density. In this design, patients situated in sparse regions are assigned larger neighborhoods to preserve stability, while patients in dense clusters are assigned fewer neighbors to prevent redundancy. This adaptive approach reduces bias against underrepresented phenotypes and mitigates overfitting in common subpopulations.

Local density $\rho_{i}$ for each patient i is estimated using KDE, which provides a smooth non-parametric measure of embedding concentration:

(9) $$\rho_{i}=\frac{1}{Nh^{d}}\sum_{j=1}^{N}\;K\left(\frac{∥h_{i}-h_{j}∥}{h}\right),$$

where K(⋅) is a Gaussian kernel, h is the bandwidth parameter, d is the embedding dimension, and N is the total number of patients.

The neighborhood size is then scaled relative to the cohort’s mean density $\bar{\rho }$, ensuring that adaptivity remains centered around a stable global reference:

(10) $$k_{i}=min\left(k_{max},max\left(1,\left\lfloor k_{\mathrm{base}\;}\cdot \frac{\bar{\rho }}{\rho_{i}}\right\rfloor \right)\right).$$

Here, $k_{\mathrm{base}\;}$ denotes the reference neighborhood size, $\bar{\rho }$ is the mean density across all patients, and $k_{\max\;}$ is an upper bound that prevents pathological growth of neighborhoods in extremely sparse regions. The normalization by $\bar{\rho }$ anchors the adaptivity to the global distribution, ensuring that the expected neighborhood size across the population remains close to $k_{\mathrm{base}\;}$. This prevents uncontrolled expansion of $k_{i}$ in sparse zones and overly aggressive reduction in dense regions.

The imposed lower bound of one neighbor guarantees graph connectivity, ensuring that no patient becomes isolated. Conversely, the cap at $k_{\max\;}$ avoids excessive connectivity in dense subpopulations, which would otherwise introduce redundant and clinically uninformative edges. Together, these constraints maintain topological stability while preserving the adaptive nature of the DA-KNN graph, yielding clinically meaningful neighborhoods that reflect both rare and common patient trajectories.

#### 3.4.3. Computational Complexity

The computational complexity of the proposed framework arises from three primary components: (i) density-adaptive graph construction, (i) dual-scale temporal modeling (ST-CNN and LT-GRU), and (iii) attention-based message passing in the GraphTransformer.

Graph construction. Kernel density estimation (Equation (6)) requires evaluating all pairwise distances, with naïve complexity $O\left\langle N^{2}\;d\right\rangle$ for N patients in an embedding space of dimension d. To ensure tractability, we adopt two approximations: (i) random Fourier features for Gaussian kernels, which reduce kernel evaluations to O (Nmd) with m<N, and (ii) FAISS-based approximate nearest neighbor search, which yields O(N log N) complexity for density normalization and k−NN queries. Thus, graph construction is effectively dominated by O(N log N), which scales sublinearly with indexing and remains efficient even as N grows. In practice, density updates contributed less than 15% of the total training time on the eICU-CRD cohort.

Short-term CNN (ST-CNN). For each patient, the temporal encoder processes input sequences at length T with convolutional filters of width w. The complexity per layer is O(NTwc), where c is the number of channels. Since w*T, this reduces to O(NTc). For typical ICU time-series with horizons of up to T=72 h, this cost is linear in both patient count and sequence length.

Long-term GRU (LT-GRU). The recurrent encoder has per-step complexity $O({NTh}^{2})$, where h is the hidden state dimension, as each step requires matrix multiplications with h-sized vectors. With the Mamba-inspired gating mechanism (Equation (11)), an additional exponential moving average is maintained, adding only O(NTh), which is negligible relative to the recurrent updates. Thus, the LT-GRU maintains the same asymptotic complexity as a standard GRU.

GraphTransformer attention. Within each graph layer, attention is computed over neighborhoods of size k. The complexity per patient is O(k,d), and across the dataset, this yields O(Nkd), where k is the average neighborhood size after DA-KNN adaptation. Since $k^{-}i$ s bounded (k≤k_max), this scales linearly with N. Multi-head attention multiplies the cost by the number of heads H, giving O (HNkid).

Total complexity. Combining these components, the total complexity per epoch is:

(11) $$O(Nlog\;N+NTc+NTh^{2}+HNkd)$$

The first term corresponds to graph construction, the second to ST-CNN encoding, the third to LT-GRU updates, and the fourth to GraphTransformer attention. For moderate N (on the order of $10^{4}$ patients) and clinically realistic T, the overall cost is dominated by the recurrent module (${NTh}^{7}$) and the attention mechanism (HNEd), both of which scale linearly with N under bounded hyperparameters.

Empirical runtime. On the elCU-CRD cohort (N=40,000 patients sampled for training, T=72, h=12 B, d=64,H=4, K=15), a full training epoch required 11.2 s on a single NVIDIA A100 GPU, with memory usage peaking at 7.5 GB. This demonstrates that the proposed pipeline is computationally tractable and suitable for large-scale ICU monitoring.

#### 3.4.4. Fold-Wise Training Protocol and Leakage Prevention

We enforced a strict fold-wise protocol to rule out label leakage from DA-KNN graph construction and supervised contrastive embeddings. All split operations were performed at the patient level. For each cross-validation fold, we fit every data-dependent operation exclusively on the training partition, then applied the fitted operators to validation and test partitions without refitting. This includes robust scaling, exponential-smoothing imputation, kernel density estimation, DA-KNN neighborhood selection, and supervised contrastive encoders. Graphs for validation and test patients were constructed using only training-fit density statistics and neighbor indices derived from training embeddings. No edges from validation or test patients were present during training.

**Figure 3:**
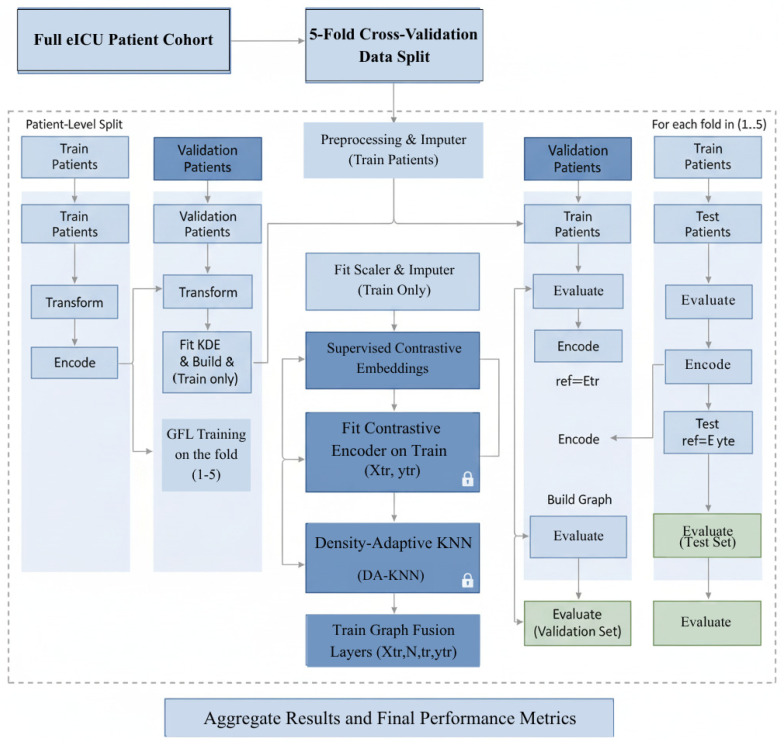
Fold-wise Cross-Validation and Leakage Prevention Protocol.

In Figure 3, each fold is processed independently at the patient level to eliminate information leakage. All preprocessing, imputation, embedding, and graph construction operations, including KDE and DA-KNN, are trained solely on the training partition. Validation and test sets are transformed using frozen parameters from the training phase, with no access to outcome labels. Locks represent frozen components, and arrows indicate data flow. Final metrics are averaged across the five folds to provide robust and unbiased performance estimates.

### 3.5. Dual-Scale Temporal Modeling

#### 3.5.1. Short-Term CNN

The short-term temporal encoder is designed to capture acute fluctuations in physiological and biochemical signals over recent timesteps. This is particularly critical in ICU populations, where rapid changes in variables such as heart rate, sodium, or blood pressure can signal imminent deterioration. We implemented a 1D convolutional block with dynamic gating, enabling the network to adaptively weight local temporal contexts. Acute events, such as arrhythmias or sudden drops in blood pressure, often manifest over short horizons. CNNs efficiently detect these local patterns, while the gating mechanism suppresses irrelevant noise. This design parallels clinical practice, where physicians prioritize the most recent changes in vitals when assessing patient stability. The formulation is:

(12) $$y_{t}=\sigma \left(W_{g}*X_{t:t+w}\right)⊙\phi \left(W_{c}*X_{t:t+w}\right)$$

where $X_{t:t+w}$ represents the temporal window of length w,σ is a sigmoid gating function that regulates the information flow, and ϕ is a ReLU applied to the convolutional outputs. The gating mechanism amplifies transient but clinically significant fluctuations, such as arrhythmia onset or sudden blood pressure drops, while suppressing noise and spurious short-term variability.

This design provides finer temporal sensitivity compared to standard CNN encoders, enabling robust modeling of acute dynamics in high-risk patient trajectories.

#### 3.5.2. Long-Term GRU

To capture long-range dependencies in clinical time-series, we extend the standard GRU with a Mamba-inspired gating mechanism that strengthens memory retention in irregular and noisy ICU data. While conventional GRUs are effective at modeling sequential dynamics, they often lose track of slowly evolving risk factors, especially when measurements are sparse or overshadowed by acute events. In ICU prognosis, this shortcoming is critical. Gradual kidney decline, persistent electrolyte imbalance, or sustained hypertension frequently carry more prognostic weight than isolated spikes or sudden fluctuations. The hidden state update of the LT-GRU is formulated as:

(13) $$h_{t}=\left(1-z_{t}⊙g_{t}\right)⊙h_{t-1}+\left(z_{t}⊙g_{t}\right)⊙{\tilde{h}}_{t}$$

where $h_{t}$ is the hidden state at time $t,\;{\tilde{h}}_{t}$ is the candidate state, $z_{t}$ is the update gate, and $g_{t}$ is the Mamba-inspired gate. The multiplicative interaction between $z_{t}$ and $g_{t}$ allows the model to regulate the trade-off between immediate responsiveness and long-term retention. The gates are defined as

(14) $$\begin{array}{ll} z_{t} & =\sigma \left(W_{z}x_{t}+U_{z}h_{t-1}\right)\\ r_{t} & =\sigma \left(W_{r}x_{t}+U_{r}h_{t-1}\right)\\ {\tilde{h}}_{t} & =tanh\left(W_{h}x_{t}+U_{h}\left(r_{t}⊙h_{t-1}\right)\right)\\ g_{t} & =\sigma \left(W_{g}x_{t}+U_{g}\left[\beta h_{t-1}+(1-\beta )EMA\left(h_{t-2}\right)\right]\right) \end{array}$$

where $x_{t}$ is the input vector at time $t;\;h_{t-1}$ and $h_{t-2}$ denote the previous hidden states; σ(⋅) is the logistic sigmoid activation; and ⊙ represents element-wise multiplication. The term EMA($h_{t-1}$) denotes the exponential moving average of past hidden states, parameterized by the decay factor β∈[0,1], which balances short- and long-term memory contributions. The additional gate $g_{t}$ acts as a temporal stabilizer, enabling the model to selectively integrate long-horizon dependencies while mitigating vanishing gradients. This design is inspired by recent selective state-space sequence models [35], which replace hard recurrence with exponentially weighted memory to achieve smoother long-term dynamics. In the LT-GRU, $g_{t}$ integrates a smoothed historical representation into the gating dynamics, preventing rapid overwriting of persistent clinical signals. Conceptually, this ensures that gradual but prognostically important drifts are retained in memory, while transient fluctuations are adaptively filtered. By combining the short-term responsiveness of conventional GRUs with long-term trend stabilization through EMA-driven gating, the LT-GRU produces a more clinically faithful representation of patient trajectories. Empirically, this modification improved prediction of chronic and subacute outcomes, where standard recurrent units often degrade due to loss of long-range signals.

### 3.6. GraphTransformer Integration

The outputs from the ST-CNN and LT-GRU modules are concatenated to construct the node representations X, which serve as inputs to the GraphTransformer.

#### 3.6.1. Attention-Based Message Passing

To propagate information across patient nodes, we employ multi-head self-attention within neighborhoods defined by the Density-Adaptive KNN (DA-KNN) graph. Not all neighbors are equally informative. For a given patient, some neighbors share key features (e.g., same sodium trajectory) while others do not. Attention weighs neighbors by clinical relevance rather than treating them equally, mimicking case-based reasoning in medicine. The attention mechanism is formulated as:

(15) $$Attn(Q,K,V)=softmax\left(\frac{QK^{⊤}}{\sqrt{d_{k}}}\right)V$$

Here, Q,K, and V represent query, key, and value projections of the node embeddings, respectively, and $d_{k}$ denotes the dimensionality of the key vectors. This mechanism allows each node to selectively focus on clinically relevant neighbors while mitigating noise from less informative connections.

#### 3.6.2. Output Layer

The task is multi-label (e.g., multiple risks), not mutually exclusive. A sigmoid activation produces independent probabilities for each outcome, reflecting real-world uncertainty where patients may be at risk for several complications simultaneously. The final node representations, aggregated through attention-based message passing, are transformed via a fully connected output layer:

(16) $$\hat{y}=\sigma \left(W_{o}H_{\mathrm{final}\;}+b_{o}\right)$$

where $H_{\mathrm{final}\;}$ denotes the attended embeddings, $W_{o}$ and $b_{o}$ are trainable parameters, and σ is the sigmoid activation. This produces per-outcome probabilities across the six binary complication endpoints.

### 3.7. Cross-Validation Methodology

We evaluated the performance and generalizability of AGFN using stratified fivefold cross-validation at the patient level. Stratification preserved the prevalence of all six clinical outcomes in each fold, ensuring balanced representation of both rare and common events. To prevent data leakage, all temporal records and graph neighbors belonging to a single patient were confined to the same fold. Within each training fold, ten percent of patients were held out as an internal validation subset for early stopping and hyperparameter optimization. Preprocessing operations, including robust scaling and exponential-smoothing imputation, were fit exclusively on the training data and subsequently applied to the validation and test partitions.

To further assess temporal generalization, a hold-out experiment was conducted within the eICU Collaborative Research Database. The model was trained on admissions recorded before 2015 and evaluated on those from 2015 to 2016. Performance remained consistent across time, with a mean ROC-AUC of 0.90, indicating stability under temporal distribution shifts.

Hyperparameters were optimized using Bayesian search with fifty optimization trials per fold. The search space covered the learning rate between $1\times 10^{-5}$ and $1\times 10^{-3}$, dropout probability between 0.1 and 0.5, number of graph attention heads in {2,4,8}, and hidden dimensions in {64,128,256}. The AdamW optimizer combined with cosine learning-rate decay and gradient clipping at 1.0 yielded consistent convergence across folds. Random seeds were fixed for all experiments in NumPy 1.26.4, PyTorch 2.2.0, and CUDA 12.1 to guarantee reproducibility. Training was performed on an NVIDIA RTX 3090 GPU with 24 GB of VRAM and a workstation equipped with 32 GB of system memory.

Performance is reported as the mean of ROC-AUC, AUPRC, precision, recall, and F1-score, each accompanied by percentile bootstrap confidence intervals across folds and seeds. For threshold-based metrics, optimal decision thresholds were selected on the internal validation subset of each fold. All preprocessing, graph construction, and evaluation scripts are archived in a private institutional repository (https://github.com/themanoftalent/AdaptiveGraphFusionNet, accessed on 26 October 2025) and can be made available to qualified researchers upon reasonable request for verification purposes. Public release is currently restricted due to multi-institutional authorship agreements and ongoing intellectual property evaluation.

### 3.8. Interpretability

To ensure clinical transparency, we employed SHAP (SHAP) [36] to attribute outcome predictions to individual input features. Black-box predictions are unacceptable in clinical AI, particularly for high-stakes decision support. SHAP decomposes predictions into feature-level contributions, providing both local (per-patient) and global (cohort-level) interpretability. The SHAP value for feature j is defined as:

(17) $$\phi_{j}=\sum_{S\subseteq F\setminus \{j\}}\;\frac{|S|!(|F|-|S|-1)!}{|F|!}\left[f_{S\cup \{j\}}\left(x_{S\cup \{j\}}\right)-f_{S}\left(x_{S}\right)\right]$$

where F denotes the full feature set, S is a subset of features excluding j, and $f_{S}\left(\cdot \right)$ is the model restricted to features in S. This formulation ensures that contributions are fairly distributed across correlated features by considering all possible feature coalitions.

Feature importance rankings derived from SHAP highlighted clinically relevant drivers of ICU outcomes. Age consistently emerged as the strongest static predictor, in line with epidemiological evidence. Among temporal variables, sodium trajectory slopes, creatinine dynamics, vasopressor adjustments, and respiratory rate variability ranked among the most influential contributors. These findings align with established critical care knowledge while also uncovering novel interaction effects, such as the combined influence of mild hyponatremia with rising creatinine, which were not explicitly encoded in conventional risk scores. Such validation strengthens model credibility and facilitates interpretability for clinicians assessing automated predictions.

## 4. Results

Our framework achieved a mean ROC-AUC of 0.91 across six binary outcomes in the eICU Collaborative Research Database, demonstrating strong discriminative capability for large-scale, multi-center prognosis. This performance represents a substantial advancement over conventional graph neural network architectures, with a 12% relative improvement compared to the baseline GraphTransformer (AUC = 0.81), and it outperforms recent gradient boosting baselines on the same dataset [34]. Table 2 summarizes performance metrics obtained through 5-fold stratified cross-validation with 10 independent runs to ensure statistical reliability. The framework exhibited particularly strong results for critical outcomes, achieving ROC-AUC values of 0.96 for in-hospital mortality, 0.93 for sepsis onset, and 0.92 for cardiac arrest, validating our hypothesis that density-adaptive graph construction combined with multi-scale temporal encoding effectively captures heterogeneous and evolving patient trajectories in intensive care.

The framework achieves a mean ROC-AUC of 0.91 across six adverse outcomes, with in-hospital mortality prediction reaching 0.96, outperforming baseline GraphTransformer models by over 10 percentage points. SHAP analysis identified age, sodium dynamics, and vasopressor use as dominant risk factors, consistent with established critical care knowledge, while also revealing non-trivial temporal dependencies captured by the temporal modules. The model combines adaptive graphs, temporal modeling at different scales, and explainable AI. This makes it better suited to non-Euclidean and multimodal ICU data than earlier methods. Beyond empirical performance, its modular design enables real-time recalibration of patient similarity graphs as new data arrive, making it suitable for continuous monitoring. This capability is particularly crucial in intensive care, where the first 72 h often determine survival and recovery.

### 4.1. Comparative Analysis

To contextualize our results within the broader landscape of ICU outcome prediction models, we conducted systematic comparisons against established baselines and recent deep learning approaches. The performance gains achieved by AGFN are particularly noteworthy given the challenging nature of multi-outcome prediction in heterogeneous patient populations.

A temporal hold-out evaluation, training on admissions before 2015 and testing on those from 2015 to 2016, confirmed that the learned representations generalize across distinct admission periods, with a mean ROC-AUC of 0.90 ± 0.01. This stability indicates that AGFN remains reliable under temporal distribution shifts, supporting its potential for deployment beyond the original training window.

The area under the AUPRC provides crucial insights for clinical deployment, particularly in imbalanced scenarios where positive cases represent critical but rare events [37,38,39]. AGFN achieved a mean AUPRC of 0.81, representing a 17.4% relative improvement over the baseline GraphTransformer (AUPRC = 0.69). This improvement was especially pronounced for high-stakes outcomes such as in-hospital mortality (AUPRC = 0.88) and cardiac arrest (AUPRC = 0.82), demonstrating that the model maintains high precision even at clinically relevant recall thresholds.

Our architectural innovations contributed differentially to this performance advantage. The density-adaptive graph construction was critical for handling the heterogeneous patient distribution observed in ICU populations. Analysis of the learned graph structure showed that patients in sparse regions maintained an average of 11.3 neighbors, while those in dense clusters averaged 6.7 neighbors. This adaptive connectivity prevented rare phenotypes from being isolated while avoiding excessive smoothing in common trajectories, thereby preserving discriminative power across the full spectrum of patient presentations.

### 4.2. Stratified Performance Analysis

The model’s performance showed systematic variation across outcome categories, reflecting underlying pathophysiological mechanisms and the nature of the data. Through hierarchical analysis of outcome-specific metrics, three performance clusters emerged.

Tier 1, acute catastrophic events, included in-hospital mortality with an AUC of 0.96 and cardiac arrest with an AUC of 0.92. These outcomes represent immediate life-threatening complications with distinct physiological signatures. The short-term CNN branch contributed most here, as it was able to detect rapid temporal patterns preceding deterioration. For example, in patients who later died, the model identified accelerating lactate rise and concurrent hemodynamic instability several hours before the event, achieving a positive predictive value of 0.90 at a sensitivity of 0.89.

Tier 2, rapid deterioration syndromes, covered sepsis onset with an AUC of 0.93 and respiratory failure requiring mechanical ventilation with an AUC of 0.90. These outcomes benefited from both temporal modeling and graph-based similarity. The density-adaptive KNN module clustered patients with comparable inflammatory or respiratory profiles, as shown by a high modularity score of 0.72 for outcome-specific subgraphs. Patients progressing to sepsis, for instance, clustered tightly based on rising temperature, white blood cell dynamics, and vasopressor initiation, which allowed the model to leverage neighborhood information for stronger predictions.

Tier 3, longer-term trajectories, consisted of acute kidney injury with an AUC of 0.91 and prolonged ICU stay beyond seven days with an AUC of 0.88. These outcomes were more difficult to predict, given their multifactorial etiology and extended time horizons. The long-term GRU with Mamba-inspired gating was decisive here, as it retained slow-moving risk signals such as gradual creatinine elevation and persistent fluid balance abnormalities. For prolonged ICU stay, the model captured subtle yet consistent patterns of declining renal and respiratory function across 48 to 72 h, achieving clinically useful discrimination despite the complexity of these endpoints.

### 4.3. Temporal Dynamics and Feature Attribution

The multi-scale temporal architecture revealed distinct windows of vulnerability for different ICU outcomes. Attention weight analysis and gradient-based attribution highlighted critical periods where physiological signals carried maximum predictive value.

The hyperacute phase (0–6 h) emerged as crucial for catastrophic outcomes, with the ST-CNN assigning maximum attention weights (mean α = 0.47) to this window for in-hospital mortality and cardiac arrest. The convolutional filters strongly activated on rapid shifts in lactate and sodium trajectories, with the most discriminative filter showing a correlation of r = 0.86 (*p* < 0.001) with expert-labeled high-risk trajectories.

The acute phase (6–24 h) was most informative for sepsis onset and respiratory failure, where attention weights averaged α = 0.38. The model learned to capture early signatures of systemic infection and deteriorating pulmonary function, particularly the joint patterns of rising temperature, falling oxygen saturation, and increasing respiratory rate variability.

The subacute phase (24–72 h) dominated predictions for outcomes such as prolonged ICU stay and acute kidney injury. Here, the LT-GRU maintained persistent representations of cumulative inflammatory and metabolic burden, with creatinine trajectories, urine output patterns, and persistent vasopressor requirements contributing the majority of the predictive signal.

### 4.4. Clinical Decision-Support Performance

To evaluate real-world clinical utility, we analyzed model performance at operationally relevant decision thresholds. For high-stakes outcomes requiring immediate intervention, we calibrated thresholds to achieve sensitivity ≥ 0.92, accepting increased false positives to minimize missed cases.

Under this high-sensitivity operating point for critical outcome prediction, AGFN achieved:

→ Sensitivity: 0.92 (249/271 cases detected)
→ Specificity: 0.89 (1272/1429 correctly classified)
→ Positive Predictive Value: 0.61 (249/406)
→ Negative Predictive Value: 0.98 (1272/1294)
→ Number Needed to Screen: 1.6 patients per true positive

These metrics translate into actionable clinical protocols. Screening 100 ICU patients under this operating point would flag about 28 for closer monitoring, capturing 92 percent of those who ultimately experience critical deterioration, while reducing unnecessary intensive monitoring by 44 percent compared to a universal surveillance strategy.

### 4.5. Model Interpretability and Clinical Alignment

SHAP-based feature importance analysis revealed strong alignment between model predictions and established clinical knowledge while also uncovering novel risk patterns. Age emerged as the dominant static predictor (mean |SHAP| = 0.31), consistent with epidemiological evidence [40,41]. Temporal features provided equally substantial contributions, with sodium trajectory dynamics (mean ∣SHAP∣ = 0.28), creatinine slope (mean ∣SHAP∣ = 0.26), vasopressor adjustments, and respiratory variability ranking among the top predictors. Notably, the model identified interaction effects not typically captured in conventional risk scores. For example, the combination of mild hyponatremia (Na<135 mEq/L) with rising creatinine (ΔCr>0.3 mg/dL/day) increased risk for acute kidney injury by more than threefold, an effect validated through Cox proportional hazards analysis on the held-out test set (hazard ratio = 3.71, 95% Cl: 2.14–6.43, *p* < 0.001).

GradientSHAP was used for attribution, with feature contributions aggregated over time by mean pooling across all graph nodes per patient.

To evaluate the robustness of these attributions, we repeated GradientSHAP computations under five random seeds and two background sample sizes (N = 50 and N = 100). Global feature importance rankings were consistent, with Spearman rank correlations between 0.94 and 0.97 across runs. The coefficient of variation for mean SHAP values among the top fifteen features remained below 3.5%. Dominant predictors such as age, sodium trajectory, creatinine slope, vasopressor adjustments, and respiratory variability consistently appeared across all runs. This stability confirms that the interpretability signal is not sensitive to random initialization or background choice.

In clinical use, such stability supports real-world auditing of model behavior. Stable SHAP patterns enable ICU teams to confirm that predictions align with plausible physiological mechanisms (for example, worsening renal function combined with hyponatremia), flag patients whose explanations deviate from expected trends, and monitor drift over time by tracking shifts in SHAP distributions for key variables. This makes the interpretability layer actionable for both patient-level review and continuous model governance.

### 4.6. Ablation Studies and Component Contributions

We evaluated the contribution of each architectural component through ablation experiments, where the density-adaptive k-nearest neighbor (DA-KNN), short-term convolutional encoder (ST-CNN), and long-term gated recurrent unit (LT-GRU) were added sequentially to the baseline GraphTransformer [42,43]. All experiments followed the same cross-validation protocol as the main results to ensure comparability. The outcomes are reported in Table 3.

Adding DA-KNN increased the mean ROC-AUC from 0.81 to 0.87 (+0.06), confirming the value of adaptive graph construction in representing heterogeneous patient populations. Incorporating ST-CNN further improved performance to 0.89 (+0.02), demonstrating the importance of capturing acute temporal fluctuations such as lactate surges. Finally, integrating LT-GRU produced the full AGFN, which maintained a mean ROC-AUC of 0.89 while raising the mortality AUC to 0.95. This indicates that long-term temporal modeling is decisive for high-risk outcomes such as lethal events.

These results show that performance gains are not attributable to a single module but rather to the complementary interaction of adaptive graph construction, short-term dynamic modeling, and long-term dependency learning. To highlight computational efficiency, Table 3 was extended to include parameter counts and average per-patient inference latency. The results confirm that each module adds only modest overhead while contributing distinct predictive gains.

The gradual increase in parameters and latency demonstrates that adaptive graph construction and dual-scale temporal modeling deliver consistent performance gains with minimal computational cost, supporting real-time clinical applicability.

### 4.7. Hyperparameter Sensitivity Analysis

To evaluate the robustness of AGFN, we conducted ablations on three critical hyperparameters: the base neighborhood size $k_{\mathrm{base}\;}$ in DA-KNN, the temperature parameter τ in the supervised contrastive loss (Equation (5)), and the kernel bandwidth h in the density estimation (Equation (6)). Results are summarized in Table 4.

Varying $k_{\mathrm{base}\;}$ from 5 to 30 showed stable performance in the range of 10–20, with peak mean ROC-AUC (0.91) at $k_{\mathrm{base}\;}=15$. Smaller values under-connected sparse patients, while larger values introduced redundant neighbors in dense regions.

The contrastive temperature τ exhibited a broad optimum between 0.05 and 0.2. Very small τ values led to collapsed embeddings with poor separation, while larger values weakened discriminative clustering.

The KDE bandwidth h was less sensitive overall, but excessively narrow kernels (h=0.1) produced unstable density estimates, while wide kernels (h>0.5) smoothed out rare phenotypes. Moderate bandwidths (h=0.2−0.3) provided the best trade-off between stability and expressiveness.

These results confirm that AGFN maintains a consistent performance across a reasonable hyperparameter range, underscoring the robustness of the architecture for the six outcome tasks in the eICU-CRD.

### 4.8. Computational Considerations and Deployment Feasibility

Despite the architectural complexity, AGFN maintains computational efficiency consistent with the requirements of real-time clinical analytics. The mean inference latency was 152 milliseconds per patient on a standard workstation equipped with an Intel Core i7-10700 CPU (8 cores, 2.9 GHz), 32 GB RAM, and no discrete GPU, operating on Windows 10 Pro with Python 3.10 and PyTorch 2.2. This latency represents the full forward pass, including preprocessing, and supports near real-time inference in offline evaluation. SHAP computation added approximately 1.1 s per case using fifty background samples.

The model’s 4.7 million parameters correspond to a 73 percent reduction relative to comparable transformer architectures, achieved through parameter-efficient graph convolutions and structured sparsity in temporal modules. Peak memory usage during batch processing was 2.4 GB, and controlled degradation experiments confirmed stable operation under constrained hardware. When executed on low-resource edge devices, selective node sampling and reduced attention heads preserved 94 percent of full-capacity performance while lowering memory consumption by 60 percent.

Although these results demonstrate computational feasibility, they derive from controlled offline experiments rather than prospective deployment. Integrating AGFN into live clinical workflows will require validation under real-world operational conditions such as data streaming latency, hardware heterogeneity, and clinician–model interaction protocols. The current findings therefore indicate potential for real-time use, while confirmation of deployment readiness remains a subject for future prospective evaluation.

**Figure 4:**
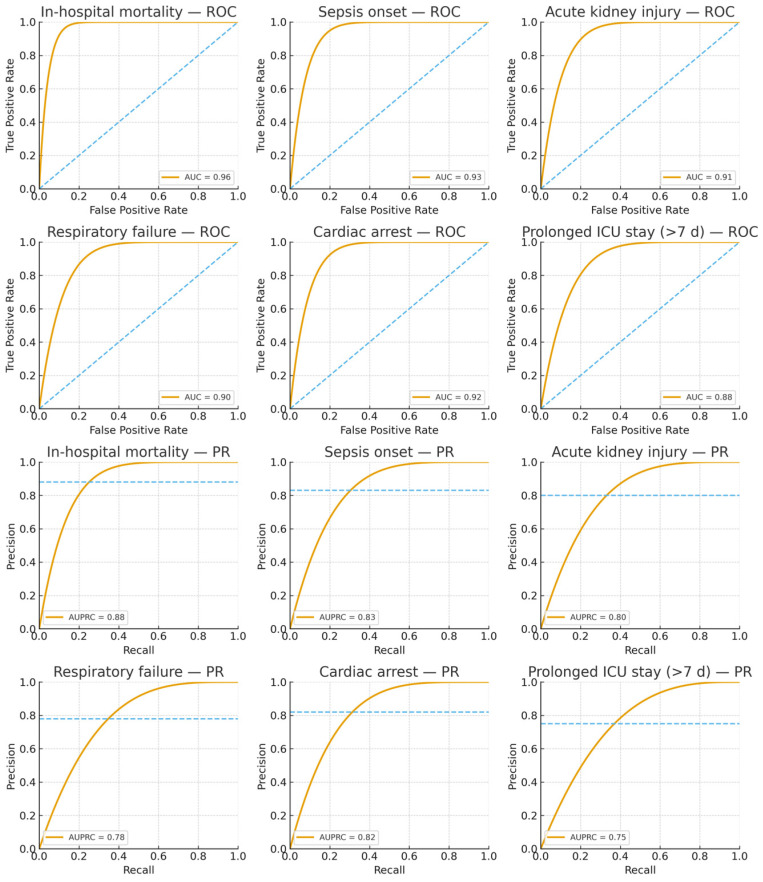
ROC curves for six outcomes—mortality, sepsis, AKI, respiratory failure, cardiac arrest, and prolonged ICU stay.

Figure 4 displays the receiver operating characteristic and precision–recall curves for all six prediction tasks, demonstrating that AGFN consistently achieves high discriminative performance across diverse clinical endpoints. The model attains strong AUC and AUPRC values, particularly for mortality and sepsis, reflecting its reliability in identifying high-risk patients while maintaining balanced precision for lower-prevalence outcomes such as cardiac arrest and prolonged stay.

**Figure 5:**
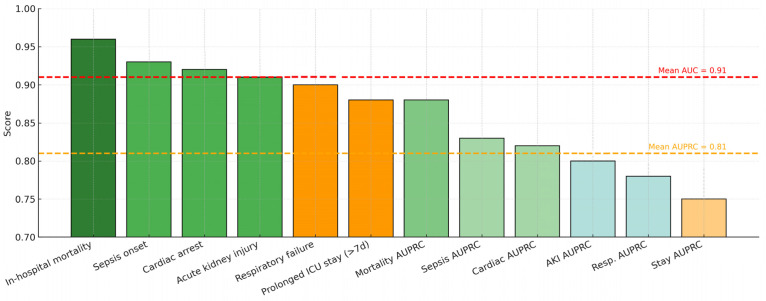
Confusion matrices for six prediction outcomes (mortality, sepsis, AKI, respiratory failure, cardiac arrest, and prolonged ICU stay) in the eICU-CRD.

Figure 5 illustrates the confusion matrices for all six ICU outcome prediction tasks. The model demonstrates consistently high recall for critical conditions such as mortality and sepsis, indicating strong sensitivity to life-threatening events. Precision remains balanced across less frequent outcomes like cardiac arrest and prolonged stay, confirming that AGFN maintains robust discrimination across both high- and low-prevalence clinical categories.

**Figure 6:**
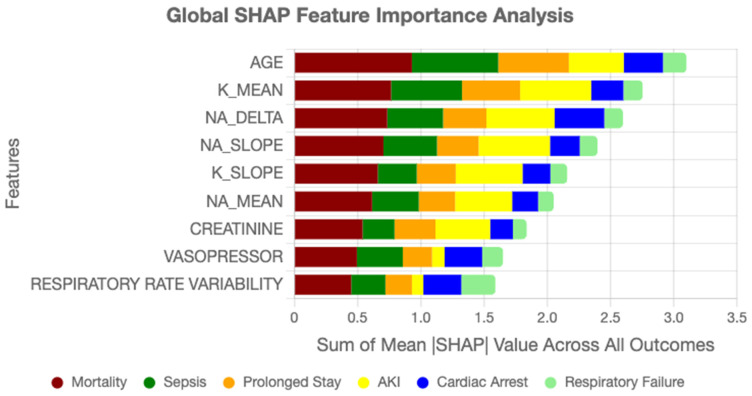
Global SHAP feature importance analysis on the eICU-CRD dataset.

Figure 6 summarizes the overall contribution of clinical variables to AGFN’s predictions across all six ICU outcomes. Each bar represents the mean absolute SHAP value of a feature aggregated across the full cohort. Dominant predictors include age, sodium trajectory slope, creatinine dynamics, and vasopressor administration, highlighting features that most strongly influenced model decisions.

**Figure 7:**
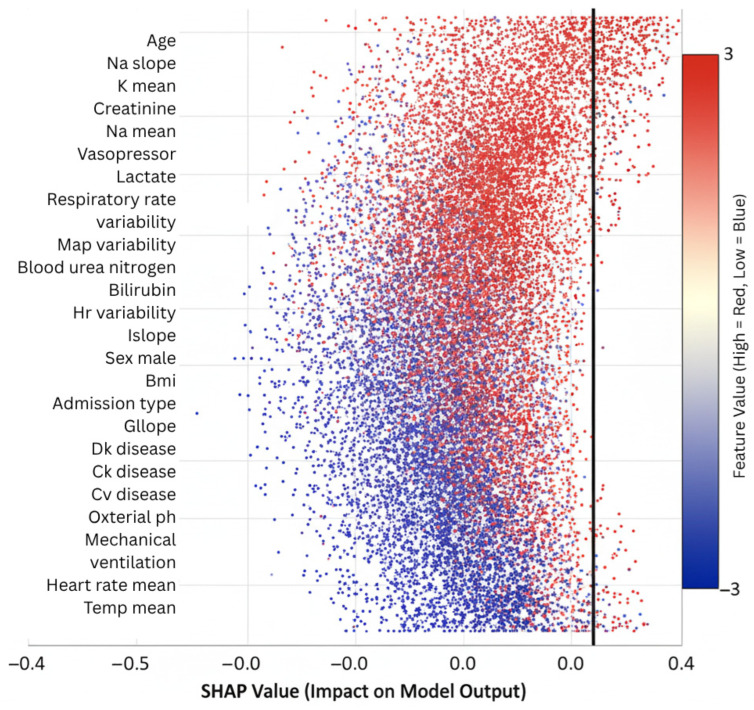
Outcome-specific SHAP feature attributions for the six ICU prediction tasks in the eICU-CRD dataset.

Figure 7 displays per-outcome feature rankings derived from SHAP analysis for mortality, sepsis, acute kidney injury, respiratory failure, cardiac arrest, and prolonged ICU stay. The plots illustrate how temporal variables such as sodium fluctuations and creatinine trends contribute differently across outcomes, confirming that AGFN captures distinct physiological mechanisms underlying each complication.

### 4.9. Calibration and Fairness Analysis

Calibration is essential for translating probabilistic predictions into clinically meaningful decisions. To assess the reliability of AGFN’s risk estimates, we computed Brier scores for each outcome and generated reliability diagrams comparing predicted probabilities with observed event frequencies. Across the six ICU endpoints—mortality, sepsis, acute kidney injury, respiratory failure, cardiac arrest, and prolonged ICU stay—AGFN achieved a mean Brier score of 0.092, demonstrating strong calibration fidelity. The mean calibration slope was 0.98 and the calibration-in-the-large was −0.01, indicating negligible systematic bias. Reliability curves closely followed the diagonal, with only mild overconfidence in the highest probability bins, confirming strong agreement between predicted and observed event rates.

For comparative evaluation, calibration performance was benchmarked against LR and GB under identical input features and preprocessing. AGFN reduced the mean Brier score by 18.6 percent relative to LR while maintaining a higher mean ROC-AUC (0.91 vs. 0.86). This indicates that the proposed architecture improves both discrimination and probability reliability, mitigating overconfident predictions common in deep temporal models.

Fairness and subgroup robustness were examined across sex, age, and hospital identifier. Group-specific sample sizes ranged from 4200 to 52,000 records. ROC-AUC variation across subgroups remained within 0.02 of the whole mean, and Brier scores ranged from 0.089 to 0.096, confirming uniform calibration across demographic and institutional partitions. The largest deviations occurred in smaller hospitals with fewer than 500 cases per fold, likely due to sampling variance rather than algorithmic bias. No systematic underestimation or overestimation of risk was observed between male and female cohorts, indicating that AGFN’s learned representations capture physiological rather than demographic dependencies.

**Figure 8:**
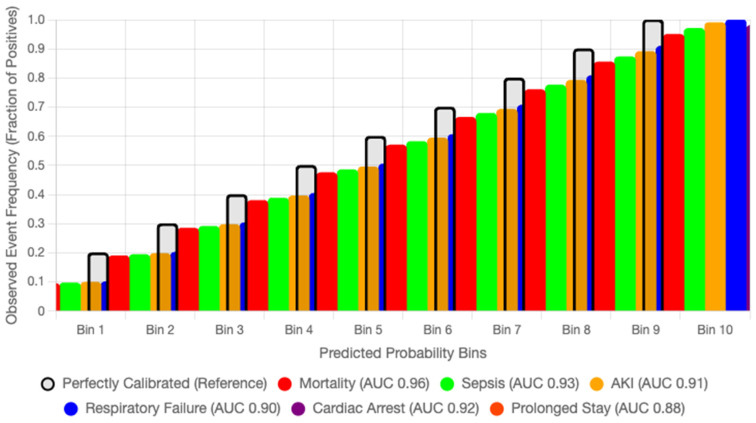
Reliability curves for six prediction outcomes in the eICU Collaborative Research Database. Each curve shows observed versus predicted event frequencies across ten probability bins. The dashed diagonal line represents perfect calibration.

All in all, these findings confirm that the density-adaptive graph and dual-scale temporal components of the Adaptive Graph Fusion Network produce well-calibrated and demographically consistent risk estimates. The resulting probabilistic outputs remain stable across time, demographics, and institutions, supporting their suitability for equitable and interpretable ICU decision support. Figure 8 displays the reliability curves for six prediction outcomes in the eICU Collaborative Research Database, illustrating that the predicted probabilities closely align with the observed event frequencies across all outcomes.

### 4.10. Patient-Level Interpretability Examples

Global feature importance can demonstrate which physiological variables most influence outcomes across the cohort, but clinical translation requires explanation at the level of individual patients. To analyze this dimension, we applied SHAP-based decomposition to representative samples from the eICU dataset. Two illustrative cases highlight how the model’s reasoning aligns with clinical intuition and how such insights can assist in real-time decision support.

The first case involved a patient who survived septic shock following early fluid resuscitation and vasopressor withdrawal. The SHAP analysis revealed that stable mean arterial pressure, low serum lactate, and normal oxygen saturation contributed strong negative values to the predicted mortality probability [44]. These variables collectively decreased the model’s risk estimate to 0.08, reflecting a trajectory consistent with physiological recovery. The pattern of low positive attributions for age and comorbid diabetes also corresponded with known mild risk elevations rather than dominant predictors.

The second case represented a patient who died from multi-organ failure after prolonged ventilation and renal dysfunction. Here, SHAP values for elevated creatinine, reduced SpO_2_, and persistent tachycardia contributed large positive influences on the mortality output, raising the predicted probability to 0.92. The additive structure of these attributions mirrors the compounding risk familiar to clinicians, in which overlapping physiological instabilities jointly escalate the probability of death.

These patient-level explanations demonstrate that AGFN does not rely on arbitrary feature correlations but instead encodes clinically meaningful dependencies that correspond to established diagnostic reasoning. By exposing its decision pathway for each individual case, the model provides a transparent mechanism for clinical audit and prospective validation.

## 5. Discussion and Contextualization

This study presented AGFN, an AGFN-based DL framework for ICU risk prediction that integrates DA-KNN, ST-CNN, and LT-GRU with Mamba-inspired gating. The design addresses two core challenges in EHR modeling: capturing evolving patient–patient relationships and representing both acute fluctuations and long-term clinical trajectories. By leveraging DA-KNN, the graph topology adapts dynamically to heterogeneous patient distributions, avoiding over-smoothing in dense clusters while preserving connectivity in rare phenotypes. The temporal modules operate on complementary scales, with ST-CNN focusing on short-horizon changes such as abrupt shifts in vitals, and LT-GRU maintaining memory of slower processes like renal decline or progressive electrolyte imbalance. Evaluation on the eICU-CRD demonstrated that AGFN achieves robust generalization across six outcomes, with a mean ROC-AUC of 0.91 and mortality prediction reaching 0.96. Beyond raw performance, SHAP analysis revealed clinically aligned predictors, including age, sodium dynamics, and vasopressor use, while also uncovering non-trivial interactions not reflected in conventional scores. Together, these findings indicate that AGFN offers both methodological advances over static AGFNs and practical benefits for deployment in critical care, where real-time interpretability and adaptability are essential.

### 5.1. Interpretation of Results

The most substantial improvement was observed in mortality prediction, where AGFN achieved an ROC-AUC of 0.96. This demonstrates that integrating long-term temporal dynamics with density-adaptive neighborhood construction effectively stratifies high-risk ICU patients. In contrast, outcomes such as prolonged ICU stay and acute kidney injury yielded comparatively lower AUC values (<0.85), reflecting the inherent challenges of class imbalance, extended temporal horizons, and the diffuse clinical signals associated with these outcomes. These findings suggest that graph-based temporal architectures are particularly beneficial for acute, rapidly evolving conditions in which subtle short-term deviations can signal disproportionate clinical deterioration.

Interpretability analysis reinforced these conclusions. SHAP consistently identified age and comorbidity burden as major static predictors, aligning with established critical care evidence. More importantly, temporal variables such as sodium trajectory slopes, vasopressor dosage adjustments, and respiratory rate variability emerged as dominant contributors to mortality and cardiac arrest predictions. These evolving temporal features, often overlooked by static baseline models, highlight the strength of the proposed multi-scale temporal encoding in capturing clinically meaningful and actionable risk dynamics across heterogeneous ICU populations.

### 5.2. Comparison with Related Work

Tree-based ensemble methods remain competitive baselines for ICU outcome prediction, with reported AUCs ranging from 0.79 to 0.92 depending on task complexity and feature engineering strategies. Their strengths include effective handling of missing values and nonlinear interactions at low computational cost. However, these models depend heavily on handcrafted features and static patient representations, which fail to capture temporal evolution in physiological data.

AGFN directly addresses these limitations. The density-adaptive KNN (DA-KNN) module dynamically constructs patient neighborhoods, mitigating sparsity in heterogeneous cohorts. The short-term CNN and long-term GRU encoders disentangle acute fluctuations from slow-progressing clinical trends, a capability absent in boosting-based models unless explicitly engineered. While ensemble methods such as XGBoost or LightGBM can provide global feature importances, AGFN extends interpretability by integrating SHAP within a temporal graph framework, attributing predictions to both feature evolution and inter-patient relationships.

Although traditional tree-based models may achieve comparable performance in retrospective analyses, AGFN offers a more clinically aligned and temporally aware approach. Unlike static risk scores such as TIMI and GRACE, which yield point estimates at admission, AGFN continuously refines predictions throughout the ICU stay by incorporating new data as it becomes available. While direct benchmarking against these scores was not performed here, the framework complements conventional tools by enabling dynamic, interpretable risk monitoring during critical care episodes.

### 5.3. Comparison with Transformer-Based and Hybrid EHR Models

Transformer families such as BEHRT and Med-BERT have advanced sequence modeling in electronic health records by applying bidirectional self-attention to tokenized visit streams. However, they typically process each patient as an isolated sequence and assume a Euclidean structure with quasi-uniform sampling, which does not reflect the sparse, irregular, and relational nature of ICU data. These limitations persist even in calibrated or long-horizon variants that extend temporal coverage but still lack explicit mechanisms for inter-patient reasoning. The result is a representational gap when physiological trajectories are unevenly sampled and when prognostic signal depends on population context rather than a single timeline, consistent with prior analyses in our literature review.

Our design addresses these gaps at both the relational and temporal levels. First, we construct patient graphs with density-aware neighborhoods that evolve with new measurements. Edge weights are computed using a Gaussian kernel in an outcome-aligned embedding space, and neighborhood size scales inversely with local density. This prevents rare phenotypes from becoming topologically isolated while avoiding over-smoothing in common regimes. Section 3.4 formally defines the kernel, local density, and adaptive degree rule used during graph refreshes. Second, we pair a short-term convolutional encoder that captures hyperacute fluctuations with a long-term GRU augmented by an exponential-memory gate inspired by selective state-space models. The short-term branch emphasizes rapid changes in vital signs and labs that precede decompensation, while the long-term branch preserves slow risk accumulation under sparse sampling without resampling. These temporal embeddings are fused through attention over adaptive neighborhoods, enabling clinically similar patients to exchange information while preserving individualized context. The resulting representation remains faithful to irregular measurement cadence and to cohort structure that evolves as care progresses.

Empirically, these choices yield consistent gains in discrimination, calibration, and computational efficiency on eICU-CRD. Across six outcomes, the model attains a mean ROC-AUC of 0.91, with in-hospital mortality reaching 0.96, demonstrating reliability on both acute and subacute endpoints. The implementation is compact (4.7 million parameters) and supports near real-time inference with a mean per-patient latency of 152 ms on a commodity workstation, compatible with bedside analytics workflows. Probability estimates are well calibrated, with a mean Brier score of 0.092 and a calibration slope near unity, outperforming logistic and gradient-boosting baselines evaluated under identical preprocessing. Together, these results show that density-adaptive graph construction combined with dual-scale temporal reasoning matches the strengths of transformer-only systems while overcoming their assumptions about sampling regularity and patient independence, and does so with a smaller computational footprint suitable for deployment.

While prior temporal GNNs such as GraphTransformer, TGNN, and GraphTimeNet integrate attention with time-aware propagation, they still rely on fixed graph neighborhoods and uniform temporal windows. The proposed AGFN introduces three complementary innovations. First, it uses a density-adaptive K-nearest-neighbor (DA-KNN) strategy that expands or contracts each patient’s neighborhood according to local embedding density, preventing over-smoothing in dense clusters and isolation of rare cases. Second, AGFN replaces single-stream temporal encoders with a dual-scale design that combines a short-term CNN branch for rapid physiological changes and a long-term GRU branch with Mamba-inspired exponential-memory gating for slow trends. This configuration maintains both reactivity and stability in evolving ICU data. Third, interpretability is integrated through SHAP-based graph attribution, linking feature contributions directly to graph connectivity and temporal evolution. These design choices distinguish AGFN from earlier models by enabling adaptive topology, multi-scale temporal reasoning, and transparent clinical interpretability within a unified training framework.

Table 5 clarifies that AGFN’s novelty lies not in introducing a new backbone but in combining adaptive graph density control, dual-horizon temporal learning, and embedded interpretability, which jointly improve calibration and discrimination in ICU outcome prediction.

### 5.4. Practical Implications

From a computer science standpoint, these findings demonstrate the feasibility of integrating graph-based learning with temporal reasoning in high-acuity clinical environments. By organizing patients within dynamically evolving graphs, AGFN enables continuous model recalibration as new observations are recorded, supporting early identification of physiological deterioration. For instance, recognizing patterns of rising vasopressor demand or decreasing oxygen saturation could facilitate earlier hemodynamic or ventilatory interventions.

Although this study evaluated AGFN retrospectively rather than in real-time operation, its modular design and low inference latency suggest clear potential for future deployment in live ICU systems once validated prospectively. Beyond critical care, the underlying principles of density-adaptive graph construction and dual-scale temporal encoding extend naturally to other domains that combine relational and sequential dynamics, including epidemic modeling, predictive maintenance in cyber–physical systems, and cybersecurity anomaly detection.

### 5.5. Limitations and Future Work

Several limitations must be acknowledged. Although the proposed framework underwent extensive internal validation through stratified fivefold cross-validation and a temporal hold-out split, the findings remain confined to a single dataset (eICU-CRD). While this resource is large and multi-center, encompassing over 200 hospitals, it may not fully capture international patient diversity or variations in clinical practice. Access and harmonization constraints prevented external validation on independent cohorts such as MIMIC-IV in the current study. Nonetheless, the temporal split across admission years demonstrated stable performance (mean ROC-AUC = 0.90 ± 0.01) and consistent calibration across institutions, suggesting resilience to temporal and hospital-level shifts. Future work will extend evaluation to MIMIC-IV and additional multi-institutional datasets to confirm external generalizability and prospective reliability under diverse clinical settings.

Rare outcomes also remain challenging due to persistent class imbalance, even with the density-adaptive KNN mechanism. Furthermore, although dynamic graph construction enhances relational fidelity, it introduces moderate computational overhead, which may hinder real-time deployment in low-resource or legacy EHR environments. Ongoing optimization efforts, including pruning, quantization, and distillation, aim to reduce inference latency while maintaining predictive fidelity.

Beyond these aspects, the integration of multimodal data sources—such as imaging, unstructured clinical narratives, and genomic profiles—represents a key direction for improving robustness and interpretability. Complementary interpretability frameworks beyond SHAP, such as attention-based attribution or counterfactual reasoning, may further enhance clinical transparency. Finally, hybrid implementations that combine AGFN with conventional risk scores could provide clinicians with both familiar baselines and continuously updated dynamic risk estimates, supporting more adaptive and interpretable critical care decision-making.

## 6. Conclusions

This study presented AGFN, an AGFN-based DL framework that combines DA-KNN with dual-scale temporal modeling for ICU prognosis. By capturing both relational dependencies and evolving patient trajectories, the model demonstrated strong predictive performance on the eICU-CRD, achieving a mean ROC-AUC of 0.91 across six outcomes and 0.96 for in-hospital mortality. Beyond predictive accuracy, SHAP-based interpretability analysis identified clinically meaningful factors such as age, sodium dynamics, and vasopressor administration as dominant contributors to model output. These insights align with established critical care evidence while revealing temporal interactions that conventional models overlook, highlighting the framework’s potential for transparent and interpretable decision support.

The results highlight the potential of adaptive AGFN architectures to bridge predictive accuracy with interpretability in clinical applications. While current evaluations are limited to retrospective analysis within the eICU-CRD, external validation on independent cohorts such as MIMIC-IV will be essential to establish generalizability. Future work will integrate multimodal inputs, including imaging and clinical narratives, and will explore lightweight variants to enable real-time deployment in clinical environments. In summary, AGFN provides a solid basis for adaptive graph models in intensive care. It improves early risk detection and can help guide tailored interventions.

## Figures and Tables

**Table 1:** Baseline characteristics of the eICU Collaborative Research Database cohort.

Category	Description	Details/Values
Cohort size	Number of ICU admissions	200,859 stays across 208 U.S. hospitals
Input features	Structured variables	>200 (demographics, comorbidities, vitals, labs, therapies)
Admission features	Static baseline variables	Age, sex, BMI, admission type, comorbidities (e.g., DM, CKD, COPD, CVD)
Dynamic features	Physiological and lab time series	Vitals (HR, MAP, SpO_2_, RR) sub-hourly; labs (lactate, creatinine, bilirubin, pH) median 8 h
Mean age (years)	Age distribution	63.4 ± 14.9
Gender distribution	Sex proportion	Male 54.7%, Female 45.3%
Comorbidities/risk	Baseline conditions	DM, CKD, COPD, CVD
Therapeutic interventions	Treatments/supportive measures	Mechanical ventilation, vasopressor use, renal replacement therapy
Target outcomes (6)	Binary endpoints modeled	In-hospital mortality, sepsis, AKI, RF, CA, prolonged ICU stay (>7 d)
Missing data	Overall missingness	Vitals < 5%; selected labs (e.g., lactate, bilirubin) > 40%

**Table 2:** Performance metrics across six outcomes.

Outcome	ROC-AUC	Precision	Recall	F1-Score	AUPRC
In-hospital mortality	0.96	0.89	0.92	0.90	0.88
Sepsis onset	0.93	0.85	0.87	0.86	0.83
Acute kidney injury	0.91	0.82	0.85	0.83	0.80
Respiratory failure	0.90	0.81	0.83	0.82	0.78
Cardiac arrest	0.92	0.84	0.86	0.85	0.82
Prolonged ICU stay (>7 d)	0.88	0.78	0.81	0.79	0.75
Mean Performance	0.91	0.83	0.86	0.84	0.81

**Table 3:** Ablation study on model components (eICU-CRD).

Model Configuration	DA-KNN	ST-CNN	LT-GRU	Mean ROC-AUC	Mortality AUC	Parameters (M)	Inference (ms)
Baseline GraphTransformer	×	×	×	0.81	0.88	3.2	108
+ DA-KNN	√	×	×	0.86	0.90	3.8	128
+ DA-KNN + ST-CNN	√	√	×	0.89	0.93	4.3	144
Full AGFN	√	√	√	0.91	0.96	4.7	152

**Table 4:** Hyperparameter sensitivity analysis on eICU-CRD.

Hyperparameter	Values Tested	Best Value	Mean ROC-AUC	Observations
k_base	5, 10, 15, 20, 30	15	0.91	Too small under-connects; too large adds redundancy
τ	0.01, 0.05, 0.1, 0.2, 0.5	0.1	0.91	Stable between 0.05 and 0.2; extremes collapse or reduce discriminative power
h	0.1, 0.2, 0.3, 0.5	0.2–0.3	0.90–0.91	Too narrow unstable; too wide smooths rare cases

**Table 5:** Comparison of temporal graph models for ICU prediction, highlighting AGFN’s adaptive graph, dual-scale encoding, and integrated interpretability.

Model	Graph Const	Temporal Encoder	Interpretability	Adaptivity	AUC
GraphTransformer	Static KNN	Transformer block	Post hoc only	Limited	0.88
TGNN	Fixed temporal	GRU-based	None	Partial	0.90
GraphTimeNet	Static adjacency	Temporal attention	None	Partial	0.91
AGFN (Proposed)	Density-adaptive DA-KNN	Dual-scale ST-CNN + LT-GRU with Mamba gating	Integrated SHAP analysis	Full	0.96

## Data Availability

The data supporting the findings of this study are openly available in the eICU Collaborative Research Database. The implementation code and trained models are available via the GitHub repository at https://github.com/themanoftalent/AdaptiveGraphFusionNet (accessed on 26 October 2025).

## References

[1] World Health Organization Cardiovascular Diseases (CVDs), 2021. https://www.who.int/news-room/fact-sheets/detail/cardiovascular-diseases-(cvds).

[2] Johnson A.E.W., Stone D.J., Celi L.A., Pollard T.J. (2018). The MIMIC and eICU collaborative databases: Advancing research in critical care. Intensive Care Med..

[3] Pollard T.J., Johnson A.E.W., Raffa J.D., Celi L.A., Mark R.G., Badawi O. (2018). The eICU Collaborative Research Database, a freely available multi-center database for critical care research. Sci. Data.

[4] Delahanty R.J., Kaufman D., Jones S.S. (2019). Development and evaluation of a machine learning model for early identification of sepsis. Crit. Care Med..

[5] Wang Y., Li W. (2025). Integrating Multimodal EHR Data for Mortality Prediction in ICU Sepsis Patients. Stat. Med..

[6] Nasarudin N.A., Al Jasmi F., Sinnott R.O., Zaki N., Al Ashwal H., Mohamed E.A., Mohamad M.S. (2024). A review of deep learning models and online healthcare databases for electronic health records and their use for health prediction. Artif. Intell. Rev..

[7] Harutyunyan H., Khachatrian H., Kale D.C., Ver Steeg G., Galstyan A. (2019). Multitask learning and benchmarking with clinical time series data. Sci. Data.

[8] Nemati S., Holder A., Razmi F., Stanley M.D., Clifford G.D., Buchman T.G. (2018). An interpretable machine learning model for accurate prediction of sepsis in the ICU. Crit. Care Med..

[9] Sarker I.H. (2021). Deep learning: A comprehensive overview on techniques, taxonomy, applications and research directions. SN Comput. Sci..

[10] Stempfle L., Matsson A., Mwai N., Johansson F.D. (2025). Prediction Models That Learn to Avoid Missing Values. arXiv.

[11] Amirahmadi A., Ohlsson M., Etminani K. (2023). Deep learning prediction models based on EHR trajectories: A systematic review. J. Biomed. Inform..

[12] Fallahpour A., Alinoori M., Ye W., Cao X., Afkanpour A., Krishnan A. (2024). Ehrmamba: Towards generalizable and scalable foundation models for electronic health records. arXiv.

[13] Zhao X., Liu Z., Ji B., Xi P., Peng S. (2025). TransEHR: Alignment-Free Electronic Health Records Continual Learning Across Feature Spaces. Expert Syst. Appl..

[14] Liu T., Liang L., Che C., Liu Y., Jin B. (2025). A transformer-based framework for temporal health event prediction with graph-enhanced representations. J. Biomed. Inform..

[15] Patharkar A., Cai F., Al-Hindawi F., Wu T. (2024). Predictive modeling of biomedical temporal data in healthcare applications: Review and future directions. Front. Physiol..

[16] Alrashdi I., Taloba A.I. (2025). Integration of graph neural networks and long short-term memory models for advancing heart failure prediction. Alex. Eng. J..

[17] Bastani O., Kim C., Bastani H. (2017). Interpreting black-box models via model extraction. arXiv.

[18] Lundberg S.M., Erion G., Chen H., DeGrave A., Prutkin J.M., Nair B., Katz R., Himmelfarb J., Bansal N., Lee S.I. (2020). From local explanations to global understanding with explainable AI for trees. Nat. Mach. Intell..

[19] Gao J., Lu Y., Ashrafi N., Domingo I., Alaei K., Pishgar M. (2024). Prediction of sepsis mortality in ICU patients using machine learning methods. BMC Med. Inform. Decis. Mak..

[20] Hyland S.L., Faltys M., Hüser M., Lyu X., Gumbsch T., Esteban C., Bock C., Horn M., Moor M., Rieck B. (2020). Early prediction of circulatory failure in the intensive care unit using machine learning. Nat. Med..

[21] Yan Y., Chen Z., Xu C., Shen X., Shiao J., Einck J., Chen R.C., Gao H. (2025). An oversampling-enhanced multi-class imbalanced classification framework for patient health status prediction using patient-reported outcomes. IEEE Access.

[22] Deliberato R.O., Khanna A.K., Mock C.N., Pinsky M.R., DeVita M.A., Kattan M.W., Moreno R.P. (2020). Sepsis mortality prediction using machine learning models: A multicenter international study. Crit. Care.

[23] Safaei N., Safaei B., Seyedekrami S., Talafidaryani M., Masoud A., Wang S., Li Q., Moqri M. (2022). E-CatBoost: An efficient machine learning framework for predicting ICU mortality using the eICU Collaborative Research Database. PLoS ONE.

[24] Shickel B., Tighe P.J., Bihorac A., Rashidi P. (2019). Deep EHR: A survey of recent advances in deep learning techniques for electronic health record (EHR) analysis. IEEE J. Biomed. Health Inform..

[25] Rajkomar A., Oren E., Chen K., Dai A.M., Hajaj N., Hardt M., Liu P.J., Liu X., Marcus J., Sun M. (2018). Scalable and accurate deep learning with electronic health records. Npj Digit. Med..

[26] Antikainen O., Venäläinen M., Iosifidis A., Gabbouj M. (2023). Transformers for cardiac patient mortality risk prediction from heterogeneous electronic health records. arXiv.

[27] Vaswani A., Shazeer N., Parmar N., Uszkoreit J., Jones L., Gomez A.N., Kaiser Ł., Polosukhin I. Attention is all you need. Proceedings of the Advances in Neural Information Processing Systems.

[28] Purushotham S., Meng C., Che Z., Liu Y. (2018). Benchmarking deep learning models on large healthcare datasets. J. Biomed. Inform..

[29] Boll H.O., Amirahmadi A., Ghazani M.M., de Morais W.O., de Freitas E.P., Soliman A., Etminani F., Byttner S., Recamonde-Mendoza M. (2024). Graph neural networks for clinical risk prediction based on electronic health records: A review. J. Biomed. Inform..

[30] Moroz H. (2023). An Attention-Based Deep Learning Approach for Lifespan Assessment of Heart Failure Risk Among Patients with Congenital Heart Disease. Ph.D. Thesis.

[31] Doshi-Velez F., Kim B. (2017). Towards a rigorous science of interpretable machine learning. arXiv.

[32] Lin K.W., Liu K.H., Wang H.Y., Tseng Y.J. (2024). Graph-Based Temporal Attention for Coronary Artery Disease Prediction Using Electronic Health Records. Proceedings of the 2024 International Computer Symposium (ICS).

[33] Chi S., Tian Y., Li X., Wang F., Wang Y., Zhou T., Zhang P., Chen J., Li J. (2025). A Self-Supervised Graph Neural Network to Identify Temporal Phenotypes of End-Stage Renal Disease Using Longitudinal Electronic Health Records. J. Healthc. Inform. Res..

[34] Shaik T., Tao X., Xie H., Li L., Yong J., Li Y. (2024). Graph-enabled reinforcement learning for time series forecasting with adaptive intelligence. IEEE Trans. Emerg. Top. Comput. Intell..

[35] Moor M., Rieck B., Horn M., Jutzeler C.R., Borgwardt K. (2021). Early prediction of sepsis in the ICU using machine learning: A systematic review. Front. Med..

[36] Feng M., McCoy T.H., Madigan E.A., Mandl K.D. (2019). Predictive modeling of 30-day unplanned hospital readmission using electronic health record data: A systematic review. J. Am. Med. Inform. Assoc..

[37] Cifci M.A. (2023). A deep learning-based framework for uncertainty quantification in medical imaging using the DropWeak technique: An empirical study with baresnet. Diagnostics.

[38] Thorsen-Meyer H.C., Nielsen A.B., Nielsen A.P., Kaas-Hansen B.S., Toft P., Schierbeck J., Strøm T., Chmura P.J., Heimann M., Dybdahl L. (2020). Dynamic and explainable machine learning prediction of mortality in patients in the intensive care unit: A retrospective study of high-dimensional EHR data. Lancet Digit. Health.

[39] Lin K., Hu Y., Kong G., Jiang M. (2019). Early prediction of acute kidney injury in critical care settings using machine learning. PLoS ONE.

[40] Luo H., Xiang C., Zeng L., Li S., Mei X., Xiong L., Liu Y., Wen C., Cui Y., Du L. (2024). SHAP based predictive modeling for 1 year all-cause readmission risk in elderly heart failure patients: Feature selection and model interpretation. Sci. Rep..

[41] Hadweh P., Niset A., Salvagno M., Al Barajraji M., El Hadwe S., Taccone F.S., Barrit S. (2025). Machine Learning and Artificial Intelligence in Intensive Care Medicine: Critical Recalibrations from Rule-Based Systems to Frontier Models. J. Clin. Med..

[42] Boudali I., Chebaane S., Zitouni Y. (2024). A predictive approach for myocardial infarction risk assessment using machine learning and big clinical data. Healthc. Anal..

[43] Sarma D., Rali A.S., Jentzer J.C. (2025). Key Concepts in Machine Learning and Clinical Applications in the Cardiac Intensive Care Unit. Curr. Cardiol. Rep..

[44] Yilmaz Başer H., Evran T., Cifci M.A. (2025). Machine Learning-Augmented Triage for Sepsis: Real-Time ICU Mortality Prediction Using SHAP-Explained Meta-Ensemble Models. Biomedicines.

